# Peripheral Vascular Trauma among Vascular Surgery Cases Operated in a Tertiary Care Hospital: A Descriptive Cross-sectional Study

**DOI:** 10.31729/jnma.6764

**Published:** 2022-01-31

**Authors:** Sushil Dahal, Robin Man Karmacharya, Amit Kumar Singh, Satish Vaidya, Prasesh Dhakal, Pratima Thapa, Prabha Shrestha, Niroj Bhandari, Sohail Bade, Sahil Bade

**Affiliations:** 1 Department of Surgery, Dhulikhel Hospital, Kathmandu University Hospital, Dhulikhel, Kavre, Nepal

**Keywords:** *Nepal*, *vascular surgical procedures*, *vascular trauma*

## Abstract

**Introduction:**

Peripheral vascular trauma can result in limb or life-threatening injuries. Early surgical intervention leads to a better outcome. Diagnosis is made clinically, by non-invasive and invasive imaging modalities. Our aim in this study is to find out the prevalence of peripheral vascular trauma among vascular surgery cases operated in a tertiary care centre of Nepal.

**Methods:**

This is a descriptive cross-sectional study of peripheral vascular injuries that underwent operative management in a tertiary care hospital of Nepal from January 2018 to May 2020. Ethical approval was taken from the Institutional Review Committee of Kathmandu University School of Medical Sciences (Registration Number 79/20). Convenience sampling technique was used. Data for the study was retrieved from operation records of the patients along with their treatment summaries and entered and analyzed in the Statistical Package for Social Sciences version 20.0. All cases with complete records were included. Conservatively managed cases and cases that underwent primary amputation were not included in the study. Point estimate at 95% Confidence Interval was calculated along with frequency and proportion for binary data.

**Results:**

Among 624 vascular surgery patients, 40 (6.41%) (4.48-8.33 at 95% Confidence Interval) patients had presented with peripheral vascular trauma during the study period. There were 26 (65%) cases where the upper limb was involved.

**Conclusions:**

The prevalence of vascular surgery for peripheral vascular trauma among vascular surgeries operated in our study was similar to other studies done in similar settings. Vascular injury needs urgent intervention and appropriate management will result in a high chance of limb salvage and survival.

## INTRODUCTION

Peripheral vascular injuries accompany up to 5% of total injuries of the extremities.^1^ Initial physiological response to vascular injury is vascular spasm.^2^ It is essential that we revascularize the limb within the golden time period, that is two hours of ischemia before there is permanent nerve damage or at most 4-6 hours after which the muscle is not salvageable.^3^ Lower limb proximal arteries are associated more with life and limb loss whereas amputation was more associated with distal arterial injuries.^4^

Mortality is associated more with penetrating injury and limb loss more with blunt injury.^4,5^ Different surgical interventions according to the type of injuries are anastomosis, lateral repair, reverse saphenous vein graft, or ligation if repair is not possible.^6^

The aim of this study is to find out the prevalence of peripheral vascular trauma among vascular surgery cases operated in the tertiary care centre of Nepal.

## METHODS

This was a descriptive cross-sectional study on the patients undergoing vascular surgery in Dhulikhel Hospital from January 2018 to May 2020. Ethical approval was taken from the Institutional Review Committee of Kathmandu University School of Medical Sciences (Registration Number 79/20). Data for the study was retrieved from operation records of the patients along with their treatment summaries. All cases with complete records were included. Conservatively managed cases and cases that underwent primary amputation were not included in the study. The sample size was calculated using the following formula:

n = Z^2^ × (p × q) / e^2^

  = (1.96)^2^ × 0.5 × (1-0.5) / (0.05)^2^

  = 385

where,

n= minimum required sample size for infinite population
Z= 1.96 at 95% Confidence Interval (CI)
p= prevalence of vascular trauma among vascular surgery cases operated in a tertiary care center taken as 50% for maximum sample size calculation
q= 1-p
e= margin of error, 5%

From that the sample size obtained is 385. Taking 10% for missing data in the hospital records, the sample size will be 428. However, the total sample size of the study is 624.

Diagnosis of vascular injury was made via clinical suspicion, examination, and adjunctive imaging methods. Hard signs (observed pulsatile bleeding, arterial thrill, bruit, distal ischemia, visible expanding hematoma) and soft signs (haemorrhage, decreased pulse compared to contralateral limb, bony injury, or proximity penetrating wound, neurologic abnormality) were assessed clinically.^7^ If a patient had a vascular injury in more than one location (e.g, injury to forearm and thigh), they were taken as two separate cases for analysis.

Although the nature of vascular injuries has been classified as blast injury, blunt injury, and penetrating injury in a meta-analysis, we opted to classify the injury as cut injury, laceration, and crush injury based on the intraoperative finding.^8^ The nature of trauma was classified as cut injury if the injury was "clean" and "sharp" cut as by sharp or bladed objects, laceration if the wound was irregular and the cut in the vessel was not clean. If there was an extensive injury to adjacent structures like muscle and significant damage to the vessel, then they were classified as a crush injury.^9^ Once vascular injury was clinically confirmed or there was the presence of visible profuse bleeding, after initial management in the emergency room, the patients were shifted to Operation Theatre and definitive management was done. In cases where there was suspicion of vascular injury but no confirmation, Doppler ultrasonography was done in the emergency room for confirmation. During surgery, the surgical principle of vascular injury management which includes, proximal control, distal control (usually on tourniquet) followed by tourniquet release, and vascular anastomosis was followed. If the transacted vessel could not be anastomosed, interposition reversed veins (usually great saphenous vein) or grafts were used. During the postoperative period, if there was no contraindication subcutaneous heparin and aspirin were used for three days for anticoagulation, and aspirin was continued for a month. Data Analysis was done in Statistical Package for Social Sciences Version 20.0. Point estimate at 95% Confidence Interval was calculated along with frequency and proportion for binary data.

## RESULTS

Among 624 vascular surgery patients, 40 (6.41%) (4.48-8.33 at 95% Confidence Interval) patients had presented with peripheral vascular trauma during the study period. Thirty-six (90%) were male and four (10%) were female. None of the cases had more than one site with vascular injury. There were 14 (35%) cases in 2018, 11 (27.5%) in 2019, and 15 (37.5%) in 2020 (five months).

The mean age was 33.13±12.97 years (Range 10-50). The mean age in males was 33.03±12.5 years and that in females was 34.00±21.4 years. The most common site of injury was the forearm, followed by the leg (Table 1). There were 26 (65%) cases where the upper limb was involved and 14 (35%) cases where the lower limb was involved.

**Table 1:** Location of vascular injury (n = 40).

Major area of the body	Location	n (%)
Upper limb	Arm	5 (12.5)
	Forearm	16 (40.0)
	Wrist	5 (12.5)
Lower limb	Thigh	2 (5.0)
	Leg	9 (22.5)
	Foot	3(7.5)

**Figure 1:**
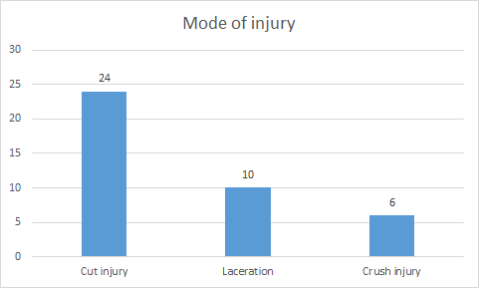
Bar diagram showing number of cases with different modes of injury.

In the mode of injury, the cut injury was most common followed by laceration and then by crush injury (Figure 1).

The mean age in cut injury cases was 36.88±12.8 years while that was 32.10±6.6 years in laceration and 19.83±15.3 years in crush injury.

The most commonly involved vessels were the ulnar artery 12 (30%) followed by radial artery 10 (25%) (Table 2). In the lower limb, the most common vessel to be involved was the anterior tibial artery (six cases, 15%).

**Table 2:** Frequency of involvement of different vessels (n = 40).

Major region of body	Blood vessel involved	n (%)
Upper limb	Brachial artery	4 (10)
	Ulnar artery	12 (30)
	Radial artery	10 (25)
Lower limb	Femoral artery	2 (5)
	Popliteal artery	2 (5)
	Posterior tibial artery	4 (10)
	Anterior tibial artery	6 (15)

More proportion of lower limb injury had laceration/ crush injury (Table 3).

**Table 3:** The number of cases in cut injury, laceration / crush injury in upper limb and lower limb. (n = 40)

		Cut Injury n (%)	Laceration / Crush Injury n (%)
Site	Upper Limb	17 (42.5)	9 (22.5)
	Lower Limb	7 (17.5)	7 (17.5)

The proportion of the patients with associated injuries that had to be dealt with during the first surgery was 18 (45%). The most common injury was an injury to adjacent bone 8 (20%) followed by injury to tendon 6 (15%) and injury to nerve+tendon 4 (10%) (Table 4).

**Table 4:** Frequency of associated injury that had to be addressed in the primary surgery (n = 18).

Associated injury	n (%)
Bone	8 (20)
Tendon	6 (15)
Nerve and Tendon	4 (10)

Two of the cases (5%) of vascular trauma operated upon underwent failure and required amputation of the affected limb owing to complications within a week of the primary operation. Both these cases had crush injuries. All the cases including the ones requiring amputation survived.

## DISCUSSION

Vascular trauma is associated with about 5% of trauma to extremities which are one of the major causes of limb and life loss if not intervened early, even with surgical intervention depending upon the nature of the injury.^10,11^ Our study shows the prevalence of vascular trauma to be 6.41% among the vascular surgeries done at a tertiary care hospital of Nepal. Ever-increasing trauma cases in Nepal is a growing public health concern.^12,13^ Our study shows more cases of peripheral vascular trauma in males. Male predominance is seen in many other studies.^12,14-17^

In a study done in India, the incidence of vascular injury in the pediatric population was 0.6% and that in the geriatric group was 0.7%; and peripheral vascular injury accounts for about 40-75% of vascular injury in India.^17^ In a study done at our center and nearby outreach centers show that fall injury and road traffic accidents were the major cause of physical trauma with male predominance, maximum in the age group 15 to 49 years and with extremity injuries 59.8%. More trauma cases were recorded in the month of October when the national festival Dashain was celebrated.^14^ In one study, however, more trauma was observed during the summer season compared to winter.^18^ We have not divided our cases according to seasons. The mean age in our series was 33.13 years. The mean age in a study by Shah, et al. as well as Sah , et al. was 35 years, very similar to our series.^12,16^

We divided the injuries into cut injury, laceration injury, and crush injury. Cut injury was a more common mode then crush and laceration injury. Crush injury was more among younger patients in comparison to cut and laceration injury. In a review by Huber, et al. vascular trauma was divided as blunt, penetrating, or combination. In a meta-analysis that was divided into the blast, blunt and penetrating trauma.^8^

In our study injuries in the upper limb are more common than in the lower limb. The ulnar artery was mostly involved 12 (30%) followed by the radial artery 10 (25%). In the lower limb, the most common vessel to be involved was the anterior tibial artery 6 (15%). In a study done at Bhairahawa, Nepal among 12 patients sustaining major vascular injuries, nine were of upper extremities and three were lower extremities. The most common vessel to be injured was the brachial artery (33.33%) followed by radial (25%), femoral (25%), ulnar artery (8.33%), and popliteal artery (8.33%).^12^

In our study, the most common associated injury was an injury to adjacent bone (20%) followed by injury to tendon (15%) and injury to nerve+tendon (10%) among 18 (45%) of cases. A study by HK Aduful and WM Hodashi in Accra regarding peripheral vascular injuries showed brachial artery injuries as the most common injury with associated nerve injuries (25% of total cases). The intervention was successful in 84.6% of patients and 7.7% of patients who required major limb amputation and an equal number of patients died from other injuries.^19^ In a study by Shah S et al, 10% of the patient had amputations.^12^ Salvage outcome in a study by Sah B, et al. was 98%.^16^ Two of the operated cases in our study underwent amputation later. A study shows amputation rates in blast injury was 19%, that in blunt injury was 16% and that in penetrating injury was 5%.^8^ Amputation rate was affected by mode of injury by high energy firearm injury, mangled extremity severity score [MESS], delayed treatment of venous injuries, and associated fractures.^20^

A study done in Malaysia among 45 peripheral vascular injuries patients shows a salvage rate of 88.9%, only six patients underwent delayed revascularization after 24 hours of injury. This study suggested that early revascularization of peripheral vascular injuries is crucial but delayed revascularization with a bypass graft of injured vessel can also have favorable salvage outcomes considering it reduces muscle ischemia and improves limb perfusion.^21^ Likewise, in a study done in India among 61 patients, the salvage rate was 90.1% even though the meantime from injury to treatment was 21 hours.^22^ Aim of surgical intervention for such trauma is continuity of distal blood flow unless there is a significant risk of reperfusion injury or if there are other pathways of blood flow.^23^

A study on peripheral arterial injuries among children in Pakistan showed more involvement of the upper limb than ours. Brachial, radial, and superficial femoral were the most involved vessels. Presentation after six hours of injury, blunt injury associated with soft tissue loss and bone fracture, involvement of distal vessels, MESS >7, and associated life-threatening injuries (e.g. head injury) were the risk factors for limb loss. The arterial repair method did not influence the rate of secondary amputation in the study. The secondary amputation rate was 7.4% whereas it is 5% in our study.^17^

Our study could not include all the cases of vascular trauma presented to our center and focused only on cases requiring operative intervention. As this is a descriptive cross-sectional study, no associations can be made and the findings are limited to the center where the study is conducted.

## CONCLUSIONS

The prevalence of vascular surgery for peripheral vascular trauma among vascular surgeries operated in our study was similar to other studies done in similar settings. The vascular injury needs an urgent intervention so as to save limb as well as life. If properly managed in coordination with the vascular surgical team, surgical intervention for vascular injury has a very high chance of limb survival.
